# The Revision of Lichen Flora Around Maxwell Bay, King George Island, Maritime Antarctic

**DOI:** 10.1007/s12275-023-00015-x

**Published:** 2023-02-27

**Authors:** Jae Eun So, Josef P. Halda, Soon Gyu Hong, Jae-Seoun Hur, Ji Hee Kim

**Affiliations:** 1 Division of Life Sciences, Korea Polar Research Institute, Incheon, 21990 Republic of Korea; 2 Department of Polar Sciences, University of Science and Technology, Incheon, 21990 Republic of Korea; 3 Museum of Orlické Hory, Jiráskova 2, Rychnov Nad Kněžnou, 516 01 Czech Republic; 4 Korean Lichen Research Institute (KoLRI), Sunchon National University, Suncheon, 57922 Republic of Korea

**Keywords:** Lichen, Lichen taxonomy, Antarctic lichen diversity, Ecological traits

## Abstract

Since the floristic study of lichens at the Barton and Weaver Peninsulas of King George Island in 2006, there have been intense investigations of the lichen flora of the two peninsulas as well as that of Fildes Peninsula and Ardley Island in Maxwell Bay, King George Island, South Shetland Islands, maritime Antarctic. In this study, a total of 104 species belonging to 53 genera, are identified from investigations of lichens that were collected in austral summer seasons from 2008 to 2016. Phenotypic and molecular analyses were incorporated for taxonomic identification. In particular, 31 species are found to be endemic to the Antarctic and 22 species are newly recorded to the Maxwell Bay region. *Lepra dactylina*, *Stereocaulon caespitosum*, and *Wahlenbergiella striatula* are newly recorded in the Antarctic, and the previously reported taxon *Cladonia furcata* is excluded from the formerly recorded list due to misidentification. We also provide ecological and geographical information about lichen associations and habitat preferences.

**Supplementary Information:**

The online version contains supplementary material available at 10.1007/s12275-023-00015-x.

## Introduction

Lichens are symbiotic organisms that are composed of lichenized fungi (mycobiont) and microalgae and/or cyanobacteria (photobiont). They have successfully adapted to severe environmental conditions that prevail in tropical, desert, high alpine, and polar regions. Lichens usually inhabit ice-free areas (Singh et al., 2018; Smith, 1988), and they are the main vegetation in the Antarctic terrestrial ecosystems along with two vascular plants, *Deschampsia antarctica* Desv. and *Colobanthus quitensis* (Kunth) Bart, and diverse bryophytes (Ochyra et al., 2002; Øvstedal & Smith, 2001).

Since 380 lichen species in Antarctica were reported in the comprehensive taxonomic survey conducted by Øvstedal and Smith (2001), the number of species has steadily increased with continuous addition of new species (Alstrup & Søchting, 2011; Halici et al., 2022; Øvstedal & Smith, 2009, 2011; Øvstedal & Schaefer, 2013; Park et al., 2018). In particular, King George Island (South Shetland Islands, Antarctica) was the subject of extensive research (Olech, 2004; Piñeiro et al., 2012; Redón, 1985; Spielmann & Pereira, 2012). Olech (2004) reported 294 taxa from the island, which represents about 77% of the lichen diversity of Antarctica as a whole.

King George Island is one of the most appropriate places for studying the biodiversity and evolution of lichenized fungi of Antarctica because of its geographical location and the diversity of lichens (Kim et al., 2006; Lee et al., 2008; Øvstedal & Smith, 2001). Floristic notes on lichens in 1993 covering the Fildes Peninsula of King George Island and Harmony Cove of Nelson Island indicated 198 species; however, determination of many of these species is unreliable due to the unavailability of the type collections or a lack of adequate collections (Hertel, 1988; Inoue, 1991, 1993). Information on the lichen species in Antarctica remains insufficient for comprehensive ecological studies despite its significance (Kim et al., 2006). Floristic research has been conducted in some of the localities (Admiralty Bay, Elephant Bay, and Lions Rump area) of King George Island (Olech, 2002); however, the areas around Maxwell Bay have often been excluded. Kim et al. (2006) reported 62 lichenized fungi from Barton Peninsula and Weaver Peninsula, part of Maxwell Bay region of the island. They conducted intensive survey close to the King Sejong Korean Antarctic Research Station in Barton Peninsula to investigate the presence and distribution of vegetation. In addition, the phylogenetic analyses of 50 lichenized fungi from King George Island were analyzed using the partial large subunit ribosomal DNA sequences (Lee et al., 2008).

However, the information on lichen diversity is insufficient due to the cryptic diversity and the less visible characteristics of microlichen species in this region.

This study aims to provide complementary lichen floristic information to understand the terrestrial biodiversity of the maritime Antarctic region and to conduct comparative studies with the biodiversity of other Antarctic Conservation Biogeographical Regions (Terauds & Lee, 2016). We investigated lichen flora intensively in the Maxwell Bay region, i.e. Barton Peninsula, Fildes Peninsula, Weaver Peninsula, and Ardley Island in King George Island during an Antarctic expedition from 2008‒2016. We also provide ecological and geographical information of collected specimens along with taxonomic identification. The past specimens from the region were re-examined as well.

## Materials and Methods

### Study Area

**Fig. 1:**
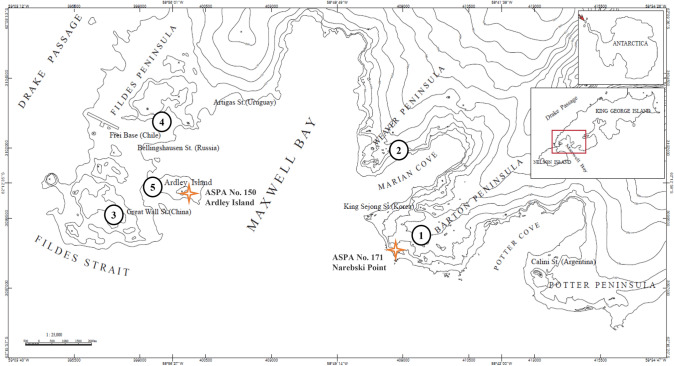
Maxwell Bay, King George Island, Antarctica. Lichen samples were collected from (1) Barton Peninsula, (2) Weaver Peninsula, (3, 4) Fildes Peninsula, and (5) Ardley Island. Antarctic Specially Protected Area (ASPA) No. 171 and ASPA No. 150 marked with asterisks

Maxwell Bay (62°25′S; 58°85′W) is located between King George Island and Nelson Island in the South Shetland Islands of Antarctica. It is a common fjord-like Antarctic embayment characterized by a U-shaped deep basin. Fieldwork was carried out in Barton Peninsula, Fildes Peninsula, Weaver Peninsula, and Ardley Island located around Maxwell Bay (Fig. 1). The study area includes the Antarctic Specially Protected Areas (ASPAs) that are designated to protect environmental and scientific values such as breeding bird colonies, relatively extensive flora, and geological features under the Antarctic Treaty System. Narębski Point (ASPA No. 171) encloses penguin colonies and is located on the southeast coast of Barton Peninsula, King George Island. Ardley Island is an islet that is 1.9 km long and is located off the southwest end of King George Island. Bird colonies inhabit the area, and the whole island has been designated as ASPA No. 150 (Fig. 1).

### Specimen Collection

A total of 982 specimens were collected from the study area and were examined during Antarctic expeditions from 2008 to 2016. Ecological information such as habitats, substrata, and other ambient traits at nearby locations were recorded for all specimens. Geographic coordinates of collection sites were recorded with GPSMAP 64 s (Garmin) or Trimble R8 GNSS /Trimble HPB450 /Geoexplorer 6000 XH Handheld, (Geosystems Inc.). The specimens were then deposited at the Korea Polar Research Institute (KOPRI).

### Taxonomic Nomenclature

All the species listed in Tables 1 and 2 followed the current name on MycoBank (www.mycobank.org/) and were confirmed with Index Fungorum (www.indexfungorum.org/), and GBIF (www.gbif.org/). If the taxonomic nomenclature was controversial in those references, the synonymy and/or taxonomy opinions of MycoBank were prioritized. The broad determination of the species was primarily conducted following Øvstedal and Smith (2001) and other relevant literature (mentioned below).

**Table 1:** Recorded lichen taxa with chemical substances in Maxwell Bay region

No	Class	Order		Family	Species	Authors	Chemical substances (by TLC)
	Dothideomycetes						
1				Mastodiaceae	*Turgidosculum complicatulum*	(Nyl.) Kohlm. & E. Kohlm	Negative
2		Capnodiales		Cystocoleaceae	*Cystocoleus* aff*. ebeneus*	(Dillw.) Thwaites	Negative
	Eurotiomycetes						
3		Verrucariales		Verrucariaceae	Verrucaria dispartita*	Vain	Negative
4					***V. durietzii***	I.M. Lamb	Negative
5					V. psychrophila*	I.M. Lamb	Negative
6					***Wahlenbergiella striatula***	(Wahlenb.) Gueidan & Thüs	Negative
	Lecanoromycetes						
7		Acarosporales		Acarosporaceae	*Acarospora austroshetlandica*^†^,*	(C.W. Dodge) Øvstedal	Negative
8					A. flavocordia*	Castello & Nimis	Rhizocarpic a
9					A. macrocyclos*	Vain	Negative
10					***A. wahlenbergii***	H. Magn	Negative
11		Baeomycetales		Hymeneliaceae	*Tremolecia atrata*	(Ach.) Hertel	Negative
12				Trapeliaceae	*Placopsis contortuplicata*^†^	I.M. Lamb	Gyrophoric a
13		Caliciales		Caliciaceae	Amandinea babingtonii*	(Hook. f. & Taylor) Søchting & Øvstedal	Negative
14					***A. coniops***^†^	(Wahlenberg.) M.Choisy ex Scheid. & H.Mayrhofer	Norstictic a
15		Caliciales		Caliciaceae	A. isabellina*	(Hue) Søchting & Øvstedal	Negative
16					A. latemarginata*	Darb. Søchting & Øvstedal	Norstictic a
17					Buellia augusta*	Vain	Norstictic a
18					*B. russa*^†^*	(Hue) Darb	Norstictic a
19					*Tetramelas anisomerus*	(Vain.) Elix	Xanthons
20					T. darbishirei*	(I.M. Lamb) Elix	Negative
21					T. granulosus*	(Darb.) A. Nordin	Xanthons
22					T. grimmiae*	(Filson) Elix	Negative
23				Physciaceae	*Physcia caesia*^†^	(Hoffm.) Hampe ex Fürnr	Atranorin, zeorin
24					*P. dubia*^†^	(Hoffm.) Lettau	Atranorin
25					*Physconia muscigena*^†^	(Ach.) Poelt	Negative
26					*Rinodina olivaceobrunnea*^†^	C.W. Dodge & G.E. Baker	Pannarin
27		Lecanorales		Catillariaceae	*Alectoria corymbosa*^†^*	Hue	Atroanorin, gyrophoric a., lecanoric a
28					*Catillaria contristans*	(Nyl.) Zahlbr	Negative
29				Cladoniaceae	*Cladonia borealis*	S. Stenroos	Barbatic a., Usnic a
30					*C. carneola*^†^	(Fr.) Fr	Barbatic a., Usnic a
31					*C. chlorophaea*^†^	(Flörke ex Sommerf.) Spreng	Fumarprotocetraric a., protocetraric a
32					*C. cornuta*^†^	(L.) Hoffm	Fumarprotocetraric a., protocetraric a
33		Lecanorales		Cladoniaceae	*C. fimbriata*^†^	(L.) Fr	Fumarprotocetraric a., protocetraric a
34					***C. galindezii***^**†**^	Øvstedal	Atranorin, porphyrilic a
35					*C. gracilis*^†^	(L.) Willd	Fumarprotocetraric a., protocetraric a., atranorin
36					*C. lepidophora*	Ahti & Kashiw	Fumarprotocetraric a., usnic a
37					*C. novochlorophaea*^†^	(Sipman) Brodo & Ahti	Fumarprotocetraric a., protocetraric a., homosekikaic a., sekikaic a
38					*C. pleurota*^†^	(Flörke) Schaer	Usnic a., zeorin
39					*C. pyxidata*^†^	(L.) Hoffm	Fumarprotocetraric a
40					*C. sarmentosa*	(Hook. f. & Taylor) C.W. Dodge	Fumarprotocetraric a
41					*C. scabriuscula*	(Delise) Nyl	Fumarprotocetraric a., protocetraric a., atranorin
42					*C. squamosa*^†^	(Scop.) Hoffm	Thamnolic a., squamatic a., barbatic a
43					***C. subulata***^**†**^	(L.) Weber ex F.H. Wigg	Fumarprotocetraric a
44					***C.* cf. *weymouthii***^**†**^	F. Wilson ex A.W. Archer	Barbatic a
45				Haematommataceae	*Haematomma erythromma*^†^	(Nyl.) Zahlbr	Atranorin, stictic a
46				Lecanoraceae	Carbonea assentiens*	(Nyl.) Hertel	Atranorin, zeorin
47					*C. vorticosa*	(Flörke) Hertel	Not investigated
48					*Lecanora aspidophora*^†^*	Vain	Norrangiformic a., usnic a., zeorin
49					*L. brialmontii*^†^*	Vain	Not investigated
50					*L. elegans* f. *lucens*	Nyl	Anthraquinones
51		Lecanorales		Lecanoraceae	*L. epibryon*	(Ach.) Ach	Stictic a., atranorin, zeorin
52					*L. gerlachei*^†^*	Vain	Negative
53					*L. parmelinoides*	Lumbsch	Atranorin
54					*L. polytropa*	(Ehrh.) Rabenh	Usnic a., zeolin
55					*Lecidella carpathica*^†^	Körb	Atranorin
56					***L. wulfenii***	(Ach.) Körb	Negative
57				Parmeliaceae	***Bryoria forsteri***^**†**^	Olech & Bystrek	Negative
58					*Cetraria aculeata*^†^	(Schreb.) Fr	Lichesterinic a., protolichesterinic a
59					*Himantormia lugubris*^†^*	(Hue.) I.M. Lamb	Barbatoric a
60					*Parmelia saxatilis*	(L.) Ach	Atranorin, salazinic a
61					*Pseudephebe pubescens*^†^	(L.) M. Choisy	Negative
62					*Usnea antarctica*^†^	Du Rietz	Fumarprotocetraric a., usnic a
63					*U. aurantiaco-atra*^†^	(Jacq.) Bory	Fumarprotocetraric a., norstictic a., usnic a
64				Ramalinaceae	*Ramalina terebrata*^†^	Hook. f. & Taylor	Usnic a
65				Sphaerophoraceae	*Sphaerophorus globosus*^†^	(Huds.) Vain	Squamatic a., thamnolic a
66				Stereocaulaceae	*Lepraria borealis*	Loht. & Tønsberg	Atranorin, rangiformic, norrangiformic a
67		Lecanorales		Stereocaulaceae	*Stereocaulon alpinum*^†^	Laurer	Atranorin, lobaric a
68					***S. caespitosum***	Redinger	Atranorin, lecanoric a
69				Tephromelataceae	Tephromela antarctica*	Øvstedal	Negative
70					*Tephromela atra*	(Huds.) Hafellner	Atranorin, alectolonic a
71		Lecideales		Lecideaceae	***Bellemerea alpina***	(Sommerf.) Clauzade & Cl. Roux	Norstictic a
72					Lecidea cancriformis*	C.W. Dodge and G.E. Baker	Negative
73					Lecidea cerussata*	Hue	Negative
74					Lecidea coralligera*	Hue	Negative
75					*Lecidea lapicida*	(Ach.) Ach	Stictic a
76					Lecidea physciella*	Darb	Usnic a
77					*Porpidia austroshetlandica*	Hertel	Porphyrylic a
78		Peltigerales		Collemataceae	*Leptogium puberulum*^†^*	Hue	Negative
79				Massalongiaceae	*Massalongia carnosa*	(Dicks.) Körb	Negative
80				Pannariaceae	*Protopannaria* *austro-orcadensis*	(Øvstedal) P.M. Jørg	Negative
81					*Psoroma antarcticum*^†^	S.G. Hong & Elvebakk	Negative
82					*Psoroma hypnorum*^†^	(Vahl) Gray	Negative
83		Peltigerales		Pannariaceae	***Psoroma tenue***^†^	Henssen	Pannaric a., porpyrylic a
84		Pertusariales		Megasporaceae	***Aspicilia* aff. *aquatica***	(Fr.) Körb	Stictic a
85					*Megaspora verrucosa*	(Ach.) Hafellner & V. Wirth	Negative
86				Ochrolechiaceae	*Ochrolechia frigida*^†^	(Sw.) Lynge	Gyrophoric a., lecanoric a
87					*Ochrolechia parella*	(L.) A. Massal	Gyrophoric a
88				Pertusariaceae	***Lepra dactylina***	(Ach.) Hafellner	Atranorin, protocetraric a.,
89					*Lepra excludens*^†^	(Nyl.) Hafellner	Norstictic a
90					Pertusaria signyae*	Øvstedal	2'-O-methylperlatolic a
91		Rhizocarpales		Rhizocarpaceae	*Rhizocarpon geographicum*^†^	(L.) DC	Psoromic a., rhizocarpic a.,
92					*Rhizocarpon nidificum*^†^	(Hue) Darb	Psoromic a., rhizocarpic a.,
93		Teloschistales		Teloschistaceae	*Athallia holocarpa*	(Hoffmann) Arup, Frödén & Søchting	Anthraquinones
94					Placodium cirrochrooides*	Vain	Parietin
95					Blastenia johnstonii*	C.W. Dodge	Anthraquinones
96					Caloplaca buelliae*	Olech & Søchting	Anthraquinones
97					*Caloplaca cerina*	(Hedw.) Th. Fr	Anthraquinones
98		Teloschistales		Teloschistaceae	*Flavoplaca citrina*	(Hoffmann) Arup, Frödén & Søchting	Parietin
99					*Gondwania sublobulata*^†^	(Nyl.) S.Y. Kondr., Kärnefelt, Elix, A. Thell, J. Kim, M.-H. Jeong, N.-N. Yu, A.S. Kondr. & Hur	Parietin
100					*Placodium regale*^†^	Vain	Parietin
101					*Polycauliona candelaria*^†^	(L.) Frödén, Arup & Søchting	Anthraquinones
102					*Xanthoria elegans*^†^	(Link) Th. Fr	Anthraquinones
103		Umbilicariales		Umbilicariaceae	*Umbilicaria antarctica*^†^*	Frey & I.M. Lamb	Gyrophoric a
104					*Umbilicaria decussata*^†^	(Vill.) Zahlbr	Gyrophoric a

Bold letter, newly recorded in the region; ^†^, the specimens which were identified (confirmed) with DNA sequence. *, endemic species of the Antarctic, TLC, thin layer chromatography, a., acid

**Table 2:** Ecological traits of the species in Maxwell Bay region

No	Species	Location^a^					Sub^b^	Occ^c^	Ecological traits
		1	2	3	4				
1	*Turgidosculum complicatulum*	・	_	_	・		r	+ +	Seashore, around coastal cliffs, lichen flora abundant, around penguin colonies
2	*Cystocoleus* aff. *ebeneus*	・	・	_	_		r, s	+ +	Arid area, dead mosses on gravelly soil, overhangs
3	*Verrucaria dispartita*	・	_	_	_		r	+ +	Coastal outcrops, intertidal zone, around penguin colonies
4	*V. durietzii*	・	_	_	_		r	+	Coastal outcrops, around penguin colonies
5	*V. psychrophila*	・	・	_	_		r	+	Coastal outcrops, intertidal zone, around penguin colonies
6	*Wahlenbergiella striatula*	・	・	_	_		r	+	Coastal outcrops, intertidal zone, around penguin colonies
7	*Acarospora austroshetlandica*	・	_	_	_		r	+ +	Seashore, on volcanic rocks
8	*A. flavocordia*	・	・	_	_		r	+ +	Inland slope, dry boulders
9	*A. macrocyclos*	_	・	_	_		r	+ +	Seashore, outcrops, around penguin colonies
10	*A. wahlenbergii*	・	_	・	_		r	+	Seashore, pebbles, around lake
11	*Tremolecia atrata*	・	_	_	_		r	+ +	Inland slope, outcrops, dry boulders
12	*Placopsis contortuplicata*	・	・	・	・		r	+ + +	Inland slope, patterned ground, dry boulders
13	*Amandinea babingtonii*	・	_	_	_		r	+ +	Coastal rocks, boulders and pebbles, around lake
14	*A. coniops*	・	・	・	_		r	+ + +	Seashore, boulders and pebbles, overhangs
15	*A. isabellina*	・	_	・	_		r	+ +	Coastal rocks, boulders and pebbles, around lake
16	*A. latemarginata*	・	_	_	_		r	+	Coastal rocks, boulders and pebbles, around lake
17	*Buellia augusta*	・	・	_	_		r	+ +	Coastal rocks, boulders and pebbles, around lake
18	*Buellia russa*	_	・	_	_		r	+	Seashore, outcrops, around penguin colonies
19	*Tetramelas anisomerus*	・	_	・	_		r	+	Seashore, Coastal pebbles
20	*T. darbishirei*	・	・	_	_		r	+	Coastal rocks, boulders and pebbles, around lake
21	*T. granulosus*	・	_	_	_		r	+ +	Coastal rocks, boulders and pebbles, around lake
22	*T. grimmiae*	・	_	・	_		m, s	+ +	Coastal rocks, inland slope, around lake
23	*Physcia caesia*	・	・	_	・		r	+ +	Seashore, around coastal cliffs, lichen flora abundant
24	*P. dubia*	・	_	_	・		r	+	Seashore, around coastal cliffs, lichen flora abundant
25	*Physconia muscigena*	・	・	_	_		m, r, s	+	Inland slope, rich vegetation, rocky soil
26	*Rinodina olivaceobrunnea*	・	_	_	_		m, s	+	Inland slope, gravelly soil
27	*Alectoria corymbosa*	・	・	_	_		r, s	+ +	Seashore, Inland slope, arid area, rocky soil
28	*Catillaria contristans*	・	_	_	_		r, s	+	Inland slope, arid area, rocky soil
29	*Cladonia borealis*	・	・	_	・		m, s	+ +	Inland slope, rich vegetation, moss mat, humid area
30	*C. carneola*	・	・	_	_		m, s	+	Inland slope, rich vegetation, moss mat, humid area
31	*C.chlorophaea*	・	・	_	・		m, s	+	Inland slope, rich vegetation, moss mat, humid area
32	*C.cornuta*	・	_	_	・		m, s	+ +	Inland slope, rich vegetation, moss mat, arid to humid area, overhangs
33	*C.fimbriata*	・	・	_	・		m, s	+ +	Inland slope, rich vegetation, moss mat, humid area
34	*C.galindezii*	・	_	_	_		m, s	+ +	Inland slope, rich vegetation, moss mat, humid area
35	*C.gracilis*	・	・	・	・		m, s	+ + +	Inland slope, rich vegetation, moss mat, arid to humid area, overhangs
36	*C.lepidophora*	・	_	・	_		m, s	+	Inland slope, rich vegetation, moss mat, humid area
37	*C.novochlorophaea*	・	・	・	_		m, s	+ +	Inland slope, rich vegetation, moss mat, humid area
38	*C.pleurota*	・	・	_	_		m, s	+ +	Inland slope, rich vegetation, moss mat, humid area
39	*C.pyxidata*	・	・	_	・		m, s	+ +	Inland slope, rich vegetation, moss mat, humid area
40	*C.sarmentosa*	・	_	_			m, s	+ + +	Inland slope, rich vegetation, moss mat, humid area
41	*C.scabriuscula*	・	・	・	・		m, s	+ +	Inland slope, rich vegetation, moss mat, arid to humid area, overhangs
42	*C.squamosa*	・	・	・	・		m, s	+ + +	Inland slope, rich vegetation, moss mat, arid to humid area, overhangs
43	*C.subulata*	・	_	・	_		m, s	+ +	Inland slope, rich vegetation, moss mat, humid area
44	*Cladonia* cf. *weymouthii*	・	_	_	_		m, s	+	Inland slope, gravelly soil, arid area, overhangs
45	*Haematomma erythromma*	・	・	・	・		r	+ + +	Intertidal zone, coastal outcrops, around perch boulders of penguin colonies
46	*Carbonea assentiens*	・	_	・	_		r	+ +	Seashore, exposed outcrops and dry boulders
47	*C.vorticosa*	・	・	_	_		r	+ +	Seashore, exposed outcrops and dry boulders
48	*Lecanora aspidophora*	・	・	・	・		r	+ +	Seashore, outcrops, around penguin colonies
49	*L. brialmontii*	・	・	_	・		r, wb	+ +	Seashore, intertidal zone, pebbles
50	*L. elegans* f. *lucens*	・	_	_	・		r	+	Seashore, around coastal cliffs, lichen flora abundant
51	*L. epibryon*	・	_	_	・		m, r, wb	+ +	Coastal rocks and pebbles, inland area
52	*L. gerlachei*	・	・	_	_		r	+	Arid area, gravelly soil, overhangs, penguin colonies
53	*Lecanora parmelinoides*	・	・	_	_		r	+	Inland area, moist rock surface, around lake
54	*L. polytropa*	・	・	・	・		r	+ +	Seashore, outcrops, around penguin colonies
55	*Lecidella carpathica*	・	_	_	_		r	+	Coastal rocks and pebbles, inland area
56	*Lecidella wulfenii*	・	・	_	_		m	+	Inland slope, arid area
57	*Bryoria forsteri*	・	・	_	_		m, r	+	Inland slope, rich vegetation, moss mat, arid to humid area
58	*Cetraria aculeata*	・	・	・	・		m, s	+ +	Inland slope, rich vegetation, moss mat, humid area
59	*Himantormia lugubris*	・	・	・	・		m, r	+ + +	Inland slope, acidic rock surfaces, moss mat
60	*Parmelia saxatilis*	・	・	_	_		m	+	Seashore, rich vegetation, moss mat, overhangs
61	*Pseudephebe pubescens*	・	・	・	_		m, r	+ + +	Inland slope, rich vegetation, moss mat
62	*Usnea antarctica*	・	・	・	・		m, r, l	+ + +	Inland slope, arid to humid area, moss mat, gravelly soil
63	*U. aurantiaco-atra*	・	・	・	・		r	+ + +	Inland slope, arid to humid area, moss mat, gravelly soil
64	*Ramalina terebrata*	・	・	・	・		r	+ + +	Seashore, around coastal cliffs, lichen flora abundant
65	*Sphaerophorus globosus*	・	・	・	・		m, l	+ +	Inland slope, rich vegetation, related to *Chorisdontium aciphyllum* and *Polytrichum strictum*
66	*Lepraria borealis*	・	・	・	・		m, r, s, l	+ + +	Seashore, Inland slope, rich vegetation, moss mat, humid area
67	*Stereocaulon alpinum*	・	・	・	・		m, s	+ +	Inland slope, rich vegetation, rocky soil
68	*S. caespitosum*	・	_	_	_		m, r	+	Inland slope, outcrops, rocky soil
69	*Tephromela antarctica*	・	・	・	_		r, s	+ +	Seashore, around coastal cliffs, lichen flora abundant
70	*T. atra*	・	_	_	_		r	+ +	Seashore, inland slope, outcrops and pebbles
71	*Bellemerea alpina*	・	・	_	_		r	+ +	Seashore, siliceous rock, around lake
72	*Lecidea cancriformis*	・	_	_	_		r	+ +	Coastal rocks, overhangs
73	*L. cerussata*	_	・	_	_		r	+	Coastal rocks, around perch boulders of penguin colonies
74	*L. coralligera*	・	・	_	_		r	+ +	Widespread and frequent on rock
75	*L. lapicida*	・	_	・	_		r	+	Coastal outcrops, intertidal zone
76	*L. physciella*	・	_	・	・		r	+ +	Inland area, around lake, rocky soil
77	*Porpidia austroshetlandica*	・	_	・	_		r	+ +	Coastal outcrops, Inland slope, dry boulders
78	*Leptogium puberulum*	・	・	_	_		r	+ +	Intertidal zone, Coastal outcrops, around perch boulders of penguin colonies
79	*Massalongia carnosa*	・	_	_	・		m	+ +	Inland slope, gravelly soil, related to *Andreaea* spp.
80	*Protopannaria austro-orcadensis*	・	・	_	_		m, r, s	+ +	Humid area, gravelly soil
81	*Psoroma antarcticum*	・	・	_	_		m, s	+ +	Seashore, inland slope, rich vegetation, moss mat
82	*P. hypnorum*	・	・	・	_		m, s	+ + +	Seashore, inland slope, rich vegetation, moss mat
83	*P. tenue*	・	・	・	_		m, r, s	+ +	Seashore, inland slope, rich vegetation, moss mat
84	*Aspicilia* aff. *aquatica*	・	_	・	_		r	+ +	Coastal outcrops, lichen flora abundant
85	*Megaspora verrucosa*	・	・	_	・		m, s, wb	+	Inland area, calcareous habitats
86	*Ochrolechia frigida*	・	・	・	・		m, l	+ + +	Inland area, rich vegetation, dead moss mat
87	*O. parella*	・	・	_	_		r	+	Seashore, dry boulders
88	*Lepra dactylina*	・	_	_	_		r	+	Seashore, dry boulders
89	*L. excludens*	・	・	_	_		r	+ +	Seashore, inland slope, dry boulders
90	*Pertusaria signyae*	・	_	_	_		m, r	+	Seashore, inland slope, dry boulders
91	*Rhizocarpon geographicum*	・	_	_	_		r	+	Seashore, inland slope, patterend ground, exposed rock surface
92	*R. nidificum*	・	・	・	・		r	+ + +	Inland slope, dry boulders
93	*Athallia holocarpa*	・	_	・	_		r	+ + +	Seashore, coastal pebbles
94	*Blastenia johnstonii*	・	_	_	・		r	+	Seashore, coastal pebbles
95	*Caloplaca buelliae*	・	_	_	_		r, l	+ +	Seashore, boulders and pebbles, growing on crustose lichens
96	*C. cerina*	・	_	_	・		m, l	+ + +	Inland area, lichen flora abundant
97	*Flavoplaca citrina*	・	・	_	・		m, r, s	+	Seashore, inland area, lichen flora abundant
98	*Gondwania sublobulata*	・	・	_	_		r	+ +	Seashore, around perch boulders of penguin colonies
99	*Placodium cirrochrooides*	・	_	_	・		r	+	Seashore, around perch boulders of penguin colonies
100	*P. regale*	・	・	_	・		r	+ + +	Seashore, around coastal cliffs, lichen flora abundant
101	*Polycauliona candelaria*	・	・	・	・		r	+ +	Seashore, around coastal cliffs, lichen flora abundant
102	*Xanthoria elegans*	・	・	・	・		r, l	+ + +	Seashore, around coastal cliffs, lichen flora abundant
103	*Umbilicaria antarctica*	・	・	_	_		r	+ +	Seashore, inland area, outcrops and boulders
104	*U decussata*	・	・	_	_		r	+	Seashore, dry boulders

^a^Specimen collected spots (1, Barton Peninsula; 2, Weaver Peninsula; 3, Fildes Peninsula; 4, Ardley Island)

^b^Substrate (l, lichen; m, moss; r, rock; s, soil; wb, whalebone)

^c^Frequency of species occurrence (+ +  + , common and easy to recognize; +  + , recognizable; +,  merely found)

### Phenotypic Analysis

Morphological characteristics were examined under a microscope (Zeiss Boom Stand Stemi 2000 Stereo Microscope) and the components were analyzed by Thin Layer Chromatography (TLC). TLC analyses were performed according to standardized methods using solvent systems A and C, and Merck TLC silica gel with *Lethariella cladonioides* (Nyl.) Krog. as the control (Culberson, 1972; Orange et al., 2001).

### Molecular Analysis

Molecular phylogenetic analyses were applied to 49 lichen species that were not clearly identified by morphological and chemical characteristics. Seventy-three specimens were identified using molecular analyses and among them, we obtained nucleotide sequences from 61 specimens through this study (Table S1). The samples of thalli were preserved at -80 °C in 100% ethanol for molecular analyses. For DNA extraction, samples were washed three times with 0.85% NaCl by vigorous mixing, spin down, and discarding of the supernatant. Samples were then freeze-dried and ground into a fine powder using a Tissue lyser (QIAGEN). DNA extraction was performed according to the DNA extraction protocol of the Exgene soil DNA mini kit (GeneAll, Cat No. 114–150). ITS1-5.8S-ITS2 (ITS) were amplified using ITS1F and LR5 (Gardes & Bruns, 1993; Vilgalys & Hester, 1990) and sequenced using the ITS1F and LR5 primers by the procedures described in a previous study (Park et al., 2012).

Sequence alignments of ITS2—nucLSU rDNA were conducted using the program jPHYDIT (Jeon et al., 2005) and were manually adjusted. Ambiguously aligned sites were excluded for phylogenetic analyses. Phylogenetic trees (Fig. S1) were inferred for ITS by maximum parsimony (MP), neighbor joining (NJ) and maximum likelihood (ML). The MP tree was obtained using the Subtree-Pruning-Regrafting (SPR) (Nei & Kumar, 2000), the NJ tree was obtained using the p-distance method and the ML tree was obtained using the GTR + I + G evolutionary model of MEGA X (Lanave et al., 1984; Nei & Kumar, 2000) with search level 5, in which the initial trees were obtained by bootstrap method (1000 replicates). All positions containing gaps were treated as missing data. Genbank accession numbers of the sequences are included in Table S1. Obtained sequences were handled for the determination of sequence similarity with reference sequences from the NCBI database (https://blast.ncbi.nlm.nih.gov/).

## Result

### Lichen Flora in Maxwell Bay Region

**Fig. 2:**
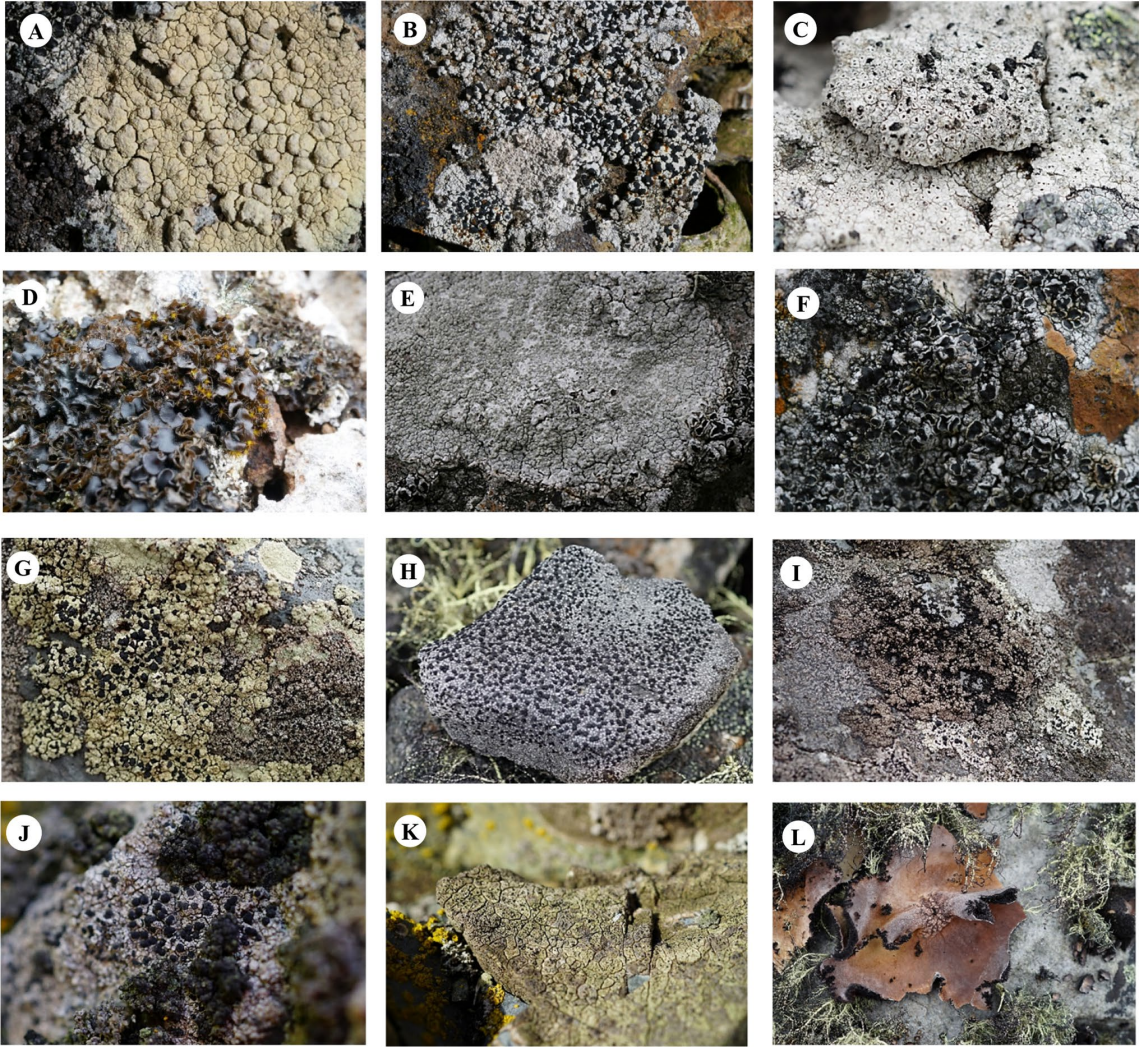
Antarctic endemic lichen species living around Maxwell Bay. **A** *Acarospora flavocordia* Castello & Nimis (HSG080116-12), **B** *Amandinea latemarginata* Darb. Søchting & Øvstedal (2016KGS-086) **C** *Buellia augusta* Vain. (2016KGS-090) **D** *Leptogium puberulu*m Hue. (2015KGS-238) **E** *Pertusaria signyae* Øvstedal (HSG080112-21), **F** *Tephromela antarctica* Øvstedal (HSG080112-19), **G** *Tetramelas anisomerus* (Vain.) Elix (2016KGIS-005), **H** *T. darbishirei* (Lamb.) Elix (2016KGS-081), **I** *T. grimmiae* (Filson) Elix (2016KGIS-004), **J** *T. granulosus* (Darb.) A. Nordin (HSG080117-09), **K** *Verrucaria psychrophila* Vain. (2016KGS-084), **L** *Umbilicaria antarctica* Frey and I.M. Lamb (2016KGS-076)

All lichen species surveyed in this study are listed under the taxonomic hierarchy in Table 1. Identification of several species was supported by molecular phylogeny in addition to morphology and chemistry. A total of one hundred-four species, belonging to 53 genera, were identified in the Maxwell Bay region (Table 1). Among them 22 species marked in bold letters are newly recorded around the bay and 31 species are endemic to Antarctica. Photographs of several Antarctic endemic species are provided in Fig. 2. Lecanorales showed the highest species diversity and followed by Caliciales and Teloschistales (Table 1).

**Fig. 3:**
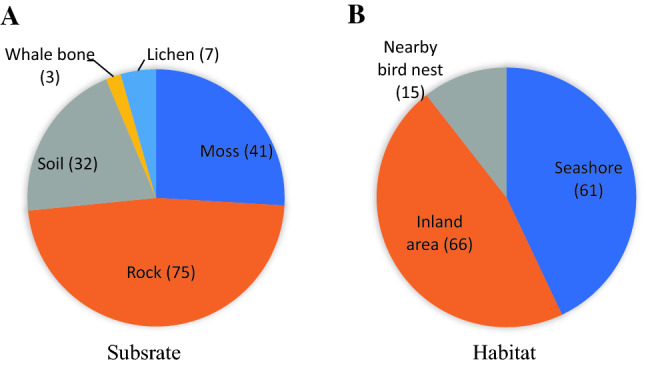
The number of species that shows each ecological trait. **A** Substrate and **B** Habitat

The observations of ecological traits were made at the time of specimen collection (Table 2). Among the sampling regions, Barton Peninsula showed the highest lichen diversity. Some species occurred on multiple substrates and habitats. Rock is the most general substrate for the lichen species in Maxwell Bay region (Fig. 3A). Seven epilichenic species were found and three crustose lichens were observed on a weathered whalebone. A few species favored several habitats: seashore, coastal cliff, and intertidal zones (Table 2). Some species showed habitat preference. Seven species were closely associated with the intertidal zone, especially the taxa of Verrucariaceae. Fifteen species were observed on perch boulders nearby nests of penguins and flying birds (Fig. 3B).

Since Kotelko and Piercey–Normore (2010) *Cladonia pocillum* (Ach.) Grognot. and *C. pyxidata* (L.) Hoffm. are conspecific by genetic evidence, only *C. pyxidata* was included in the species list (Tables 1 and 2).

### Newly Reported Taxa in Maxwell Bay Region

**Fig. 4:**
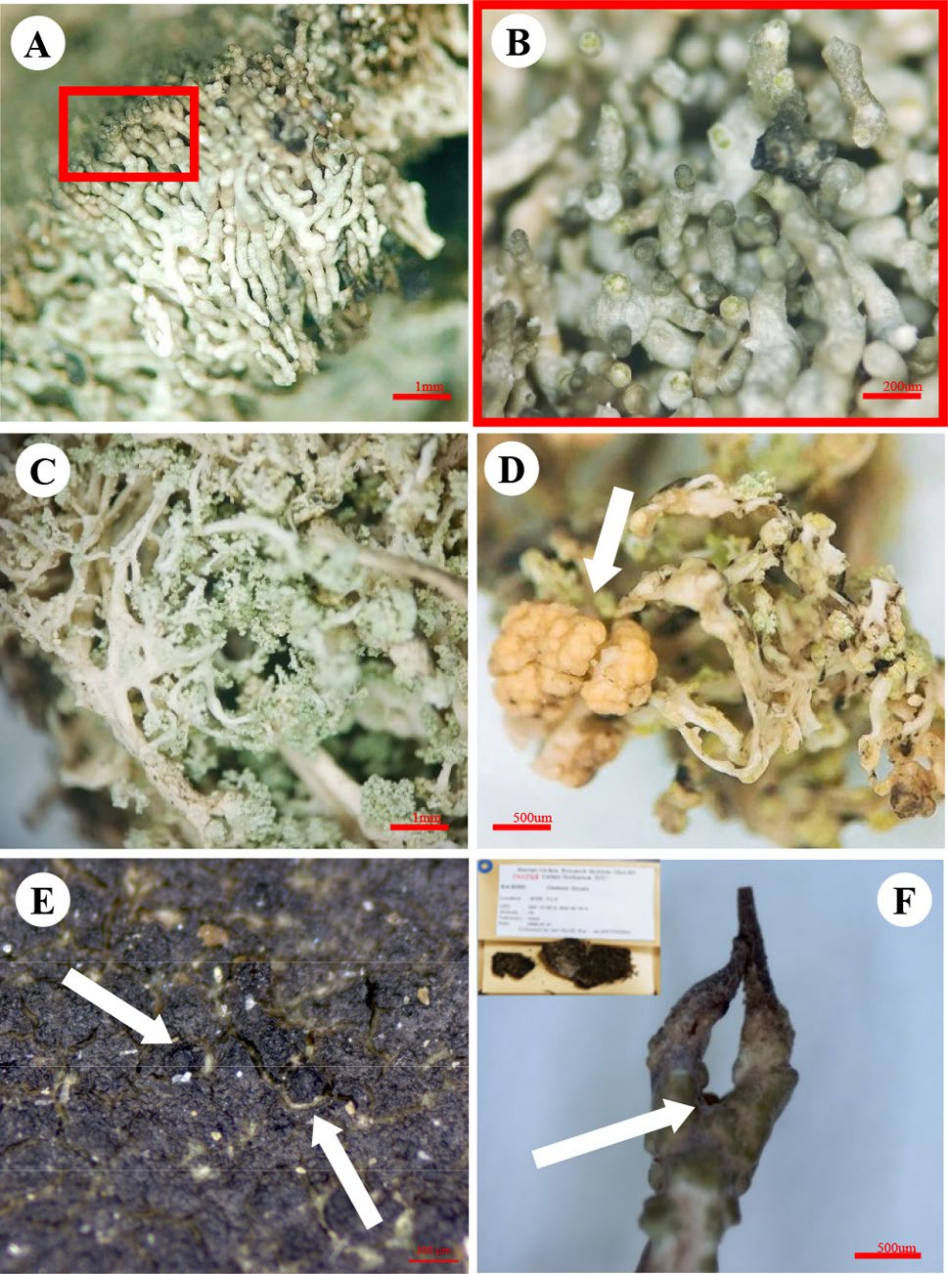
Intriguing specimens and taxa. **A**–**E**, Newly reported species from Antarctica. **A** and **B**, *Lepra dactylina* (specimen no. 2017-Ant-053). **A** Whole thallus of *L. dactylina* and **B** numerous isidia with dark-brown immersed discs. **C** and **D** *Stereocaulon caespitosum* (2017-Ant-061). **C** Pseudopodetia of *S. caespitosum* and **D** its apothecia (white arrow). **E** *Wahlenbergiella striatula* (2016KGS- 087) with perithecia (white arrow). **F** *Cladonia gracilis* (HUR ANT050826) which were previously misidentified. The closed axil (white arrow) is the diagnostic key

Twenty-two lichen species are newly recorded in Maxwell Bay region, King George Island (Table 1). The following list has arranged in alphabetical order. Illustrations of several lichen species are shown in Figs. 2 and 4.

*Acarospora flavocordia* Castello & Nimis (Fig. 2A).

Lichen thallus effigurate and areolate, yellowish, brown apothecia are common on rock. Antarctic endemic. Examined specimen: HSG080116-12.

*Acarospora wahlenbergii* H. Magn.

Lichen thallus effigurate, brownish, blackish apothecia are common on rock. Bipolar. Examined specimen: 2016KGS-127.

*Amandinea babingtonii* (Hook. f. & Taylor) Søchting & Øvstedal.

Lichen thallus subeffigurate and areolate, brownish, black apothecia are common on rock. Antarctic endemic. Examined specimen: HSG080113-31, HSG080113-32.

*Amandinea coniops* (Wahlenberg.) M.Choisy ex Scheid. & H.Mayrhofer.

Lichen thallus crustose and granulose to areolate, greyish, black apothecia are common on rock. Bipolar. Examined specimen: HSG080113-32.

*Aspicilia* aff. *aquatica* (Fr.) Körb.

Lichen thallus rimose-arelate, whitish, black apothecia suncken in thallus are common on rock. Bipolar. Examined specimen: HSG080117-08.

*Bellemerea alpina* (Sommerf.) Clauzade & Cl. Roux.

Lichen thallus areolate, blue-grey, brown apothecia sunken in thallus are growing on rock. Bipolar. Examined specimen: HSG080116-06.

*Caloplaca buelliae* Olech & Søchting.

Lichen thallus invisible, orange apothecia are growing on rocks or lichens (Especially epilichenic on *Buellia* spp.). Antarctic endemic. Examined specimen: HSG080117-09.

*Cladonia galindezii* Øvstedal.

Primary thallus squamulose and persistent, podetia brownish green without scyphi are growing on moss and soil. Antarctic endemic. Examined specimen: HL090809-15B.

*Cladonia subulata* (L.) Weber ex F.H. Wigg.

Primary thallus squamulose, podetia subulate to scyphose and farinose sorediate are growing on moss and soil. Cosmopolitan. Examined specimen: PCH080110-37, PCH110127-13, 2015KGS-053, 2016KGS-009A.

*Cladonia* cf. *weymouthii* F. Wilson ex A.W. Archer.

Primary thallus disappearing, subulate and sorediate podetia are growing on moss and soil. Cosmopolitan. Examined specimen: PCH110124-05, 2016KGIE-013.

*Lecanora elegans* f. *lucens* Nyl.

Lichen thallus crustose and lobulate, orange. Vivid orange apothecia are common on rock. Southern hemisphere. Examined specimen: ANT050906.

*Lecidella wulfenii* (Ach.) Körb.

Lichen thallus disappearing, greyish granules, black apothecia are common on moss. Bipolar. Examined specimen: HSG080116-01.

*Lepra dactylina* (Ach.) Hafellner (Fig. 4A and B).

Lichen thallus subfruticose and isidiate, whitish, immersed blackish apothecia are growing on rock. Bipolar. Examined specimen: 2017-Ant-053.

*Pertusaria signyae* Øvstedal (Fig. 2E).

Lichen thallus coralloid isidiate, greyish are growing on rock. Apothecia are uncommon. Antarctic endemic. Examined specimen: HSG080112-21, HSG080117-04.

*Psoroma tenue* Henssen.

Lichen thallus squamulose, brownish, apothecia brownish and blackish are common on moss, soil and moist rock. Cosmopolitan. Examined specimen: HSG080113-17, HSG080113-14.

*Stereocaulon caespitosum* Redinger (Fig. 4C and D).

Lichen thallus white, pseudopodetia papillae to terete forming dark brown cephalodia are growing on rocky soil. Southern hemisphere. Examined specimen: 2017-Ant-061.

*Tephromela antarctica* Øvstedal (Fig. 2F).

Lichen thallus areolate, whitish and black apothecia are growing on soil and rock. Antarctic endemic. Examined specimen: 2017-Ant-061.

*Tetramelas darbishirei* (I.M. Lamb) Elix (Fig. 2H).

Lichen thallus areolate, greyish and black apothecia are common on rock. Antarctic endemic. Examined specimen: 2016KGS-081.

*Tetramelas grimmiae* (Filson) Elix (Fig. 2I).

Lichen thallus crustose, greyish and black apothecia are common on moss and soil. Antarctic endemic. Examined specimen: 2016KGIS-004.

*Verrucaria dispartita* Vain.

Lichen thallus crustose and rimose, black dot-like perithecia are common on rocks, especially intertidal zone. Antarctic endemic. Examined specimen: 2016KGS-088.

*Verrucaria durietzii* I.M. Lamb.

Lichen thallus effigurate with lobate margin, greyish, black dot-like perithecia are common on rocks, especially intertidal zone. Southern Hemisphere. Examined specimen: HSG080112-30.

*Wahlenbergiella striatula* (Wahlenb.) Gueidan & Thüs (Fig. 4E).

Lichen thallus crustose and subgelatinous, dark green, irregular shape of perithecia are common on rocks, especially intertidal zone. Cosmopolitan. Examined specimen: 2016KGS-087.

### Delisted Species, *Cladonia furcata* (Huds.) Schrad.

*Cladonia furcata* (Huds.) Schrad. has been previously reported once in Antarctica, based on the specimens from Barton Peninsula (Kim et al., 2006). The specimens were re-examined in this study (no. HUR ANT050856 and no. HUR ANT050857; Fig. 4F). They showed a morphology that was typical of *Cladonia gracilis* subsp. *elongata* (Wulfen) Vain. An unbranched to somewhat branched podetia and closed axil were observed (Fig. 4F). Tips were pointed and seldom narrowly scyphose. Typically, open axil and numerous squamules were observed in *C*. *furcata*. Cracks on the podetia and damaged tips were frequently observed and these may have been misconceived as the ‘open axil’ trait.

**Fig. 5:**
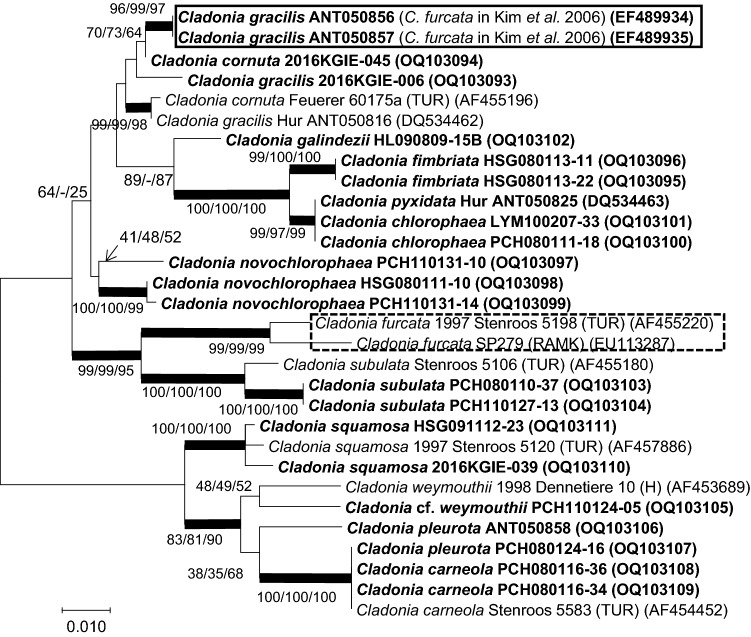
Molecular phylogeny of the genus *Cladonia* in Maxwell Bay region. The trees were obtained by Maximum Likelihood method based on ITS rDNA. Additional trees were generated by Maximum Parsimony and Neighbor joining methods, the respective support values (ML/NJ/MP) are noted. Branches supported with bootstrap values > 70 and maintained by every method are indicated in thick. The specimens identified using molecular data in this paper are bold letters. Solid box indicates misidentified specimens and dotted box shows *C. furcata*

In these specimens, only fumarprotocetraric acid was found by TLC. The acid is found in both *C. gracilis* (L.) Willd. and *C. furcata* (Ahti & Stenroos, 2013). It was also proven to be closely related to *C. gracilis* based on the molecular phylogeny (Fig. 5). We suggest the delisting of *C. furcata* from the lichen list provided in Kim et al. (2006).

## Discussion

Around the Maxwell Bay, twenty-two new lichen species have been identified (Table 1). Above all, *Lepra dactylina*, *Stereocaulon caespitosum*, and *Wahlenbergiella striatula* were previously unrecorded around the Maxwell Bay area, besides being foreign to Antarctica. *L. dactylina* was previously classified as *Pertusaria dactylina* (Ach.) Nyl. and is known as circumpolar species in the Arctic (Brodo et al., 2001), as reported by Olech (2002, 2004) from Lion Rump, King George Island; however, the records were subsequently revised to exclude this species (Olech and Slaby, 2016). Our specimen (no. 2017-Ant-053) exhibited a whitish thallus with numerous tall isidia (Fig. 4A and B), not just a thick crust, compared to the other *Pertusaria* spp. such as *P. excludens* Nyl. and *P. signyae* Øvstedal. (Brodo et al., 2001; Thomson, 1984). *Stereocaulon caespitosum* observed in New Zealand and Tasmania were flattened and had cracked pseudopodetia without enlarged tips (Smith & Øvstedal, 1991), and those collected from South Georgia had the same features. This species had been found in the subantarctic region but not in the Antarctic (Øvstedal & Smith, 2001). In this study, the specimen (no. 2017-Ant-061) collected from the Barton Peninsula was comparable to and corresponded with the descriptions of *S. caespitosum* (Fig. 4C). It should be noted that the Antarctic specimen showed large pale orange apothecia which has not been observed previously (Fig. 4D).

Gueidan et al. (2009) conducted a generic revision of Verrucariaceae. *Verrucaria striatula* was combined into the new genus *Wahlenbergiella* with *V. mucosa* according to the molecular and morphological evidence. This species is a common lichen on the littoral zone in the Northern Hemisphere but has seldom been recorded in the Southern Hemisphere. The specimen collected from New Zealand was described by Santesson (1939) and named *V. striatula* subsp. *australis*. Currently, two of the subspecies are in the New Zealand lichen flora list together as *Wahlengergiella* (de Lange et al., 2018). *Verrucaria striatula* subsp. *australis* showed a more effuse thallus margin (McCarthy, 1991) than that in the Northern Hemisphere, and our specimen shared characteristics with the former. The prominent perithecia (a small flask-shaped fruiting body that contains the ascospores) are often irregular in shape (Fig. 4E), and ostioles are comparatively large. The thallus is distinctive in its cover of black dots and ridges which gives the surface a rough texture. The combination of dark ridges and tiny black dots on a green-black thallus is a typical feature of this species. *Verrucaria amphibia* (Clemente) may be confused with *W. striatula*; however, it is darker with ridges but lacks the dot matrix that gives the thallus surface texture (Brodo & Santesson, 1997). TLC was negative in the examined specimen. *Wahlenbergiella striatula* is a pyrenolichen occurring on siliceous maritime rocks in the mid- to high-tide mark on rocky coasts. *Wahlenbergiella striatula* and *Verrucaria* spp. is usually found on the boulders, particularly near the Chinstrap penguin colonies (Table 2).

We identified 104 species of 53 genera and added 22 unrecorded species to the previous lichen floristic studies around the Maxwell Bay region (Guzmán & Redón, 1981; Hu, 1998; Inoue, 1991, 1993; Jianbin, 1996; Kim et al., 2006; Olech, 1996, 2004; Øvstedal & Smith, 2001). The Barton Peninsula showed the highest lichen diversity with 101 species among the sampling regions for the number of lichen taxa recorded. A Polish lichenologist, Olech conducted a comprehensive lichen floristic study of King George Island and 153 sampling points were visited for the study (Olech, 2004). In the study, 253 lichen species were reported, of which 74 taxa were newly recorded. Only 16% of the newly recorded taxa were found around Maxwell Bay, as most of the sampling points (101 points) were concentrated at the Admiralty Bay where the Polish Antarctic research station is located. From Barton Peninsula, Olech (2004) reported just 16 species from three points. Because of the harsh environment and remoteness of Antarctica, detailed Antarctic lichen floristic studies have been limited to areas that are near the Antarctic research stations, so that our investigation was also carried out around the Korean Antarctic Research Station. However, the topography of the Barton Peninsula is much dynamic, glacio-marine origin, and is marked by fjords, cirque, flatforms, and hanging valleys covered with bedrocks, rock fragments, moraines, raised beaches, patterned grounds, and patterned beaches (Chang et al., 2003). The complex topography of the peninsula provides a variety of habitats for diverse lichens despite the relatively narrow exposed area. This intensive survey noted that the Barton Peninsula exhibited noticeable lichen diversity and variety and may be referred to as ‘The diversity hotspot’ (Green et al., 2011; Ji et al., 2016). Given the geographical and ecological significance of the Antarctic research, the comprehensive study of the peninsula was comparatively lacking prior to this survey.

**Fig. 6:**
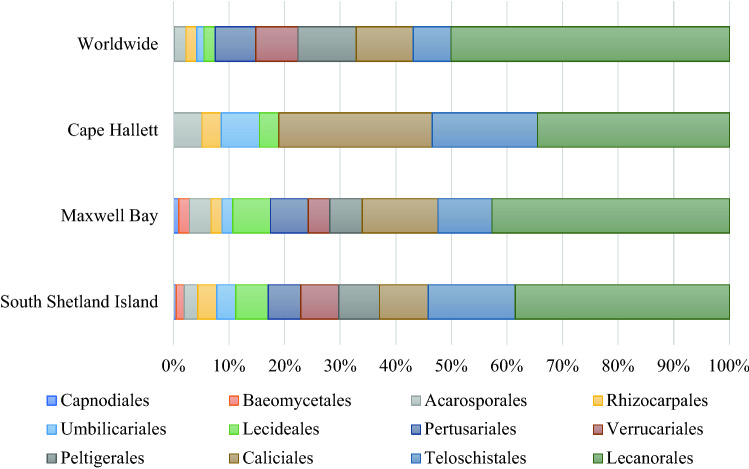
The comparison of species ratio of lichen taxonomic orders. The ratio obtained from the literatures reported species around Worldwide (Lücking et al., 2017) and Antarctic regions, Cape Hallett (Green et al., 2015), Maxwell Bay (this study), and South Shetland Islands (Øvstedal & Smith, 2001)

Our result shows that Lecanorales had the highest species diversity, followed by Caliciales and Teloschistales (Table 3 and Fig. 6). This trend was seen in other studies in the regions of King Gorge Island and South Shetlands Islands. Lecanorales is also the most diverse taxa of lichens worldwide. Ostropales and Arthoniales are highly diverse worldwide (Table 3), few have been reported from the Antarctic region because of their micro size or inconspicuous parasitic (lichenicolous) taxa and a lack of specialists; Olech (2004) only reported six taxa of Arthoniales and one of Ostropales from King George Island. On the other hand, while most diverse orders were the same, the proportions of Lecanorales and Caliciales were similar in Cape Hallett in the Eastern Antarctic region (i.e., a continental Antarctic climate region), one of the richest sites for terrestrial biodiversity in the Ross Sea region where fifty-nine lichen species were reported for eight sites (Green et al., 2015). Most species were crustose and a few were foliose near an Adelie penguin colony, which acted as a major source of ammonia (Theobald et al., 2013). Other eastern Antarctic Lichen flora reported from Schirmacher Oasis and Larsemann Hills, Prydz Bay included up to 69 and 25 lichen species respectively (Singh & Nayaka, 2017). The major lichen taxa recorded in the region consisted of *Buellia* with 10 species, followed by 9 species of *Lecanora*, and 5 species each of *Caloplaca* and *Umbilicaria*. However, owing to the diverse habitats of lichen in the Antarctic terrestrial ecosystem, we expect that more cryptic taxa will be reported in future studies, particularly, in the eastern Antarctic region where fewer studies have been conducted than the western Antarctic region.

**Table 3:** The comparison of the species diversity of lichen taxonomic orders

Order	South Shetland Island	Maxwell Bay	Cape Hallett	Worldwide
	Øvstedal and Smith (2001)	on the survey	Green et al. (2015)	Lücking et al. (2017)
Acarosporales	5	4	3	259
Arthoniales	2	0	0	1,541
Baeomycetales	3	2	0	21
Caliciales	18	14	16	1,276
Capnodiales	1	1	0	5
Lecanorales	79	44	20	6,231
Lecideales	12	7	2	249
Ostropales	2	0	0	3,261
Peltigerales	15	6	0	1,301
Pertusariales	12	7	0	907
Rhizocarpales	7	2	2	236
Teloschistales	32	10	11	841
Umbilicariales	7	2	4	168
Verrucariales	14	4	0	946
Total (species)	209	103	58	17,242

Furthermore, a significant number of unrecorded species were discovered and most of them were microlichens, which suggests that the biodiversity of the Antarctic lichens may be not to be fully revealed. Lichen flora in the Antarctic are surprisingly rich, and are equally at risk from environmental change as lichens in other regions (Chown et al., 2015). In particular, glacial outposts are worthy to be investigated for further understanding of the Antarctic floristic composition and their roles in the ecosystem in the context of global warming, and more information on the lichen diversity (as the most dominant component of Antarctic terrestrial ecosystems) is required.

## Electronic supplementary material

Below is the link to the electronic supplementary material.

Supplementary file1 (PDF 312 kb)

## Data Availability

The datasets generated and analyzed during the current study are available from the correspo nding author on reasonable request. GeneBank accession numbers of the sequences are included in Table S1.
