# Supplementary material for: The Revision of Lichen Flora Around Maxwell Bay, King George Island, Maritime Antarctic
Source: J Microbiol. 2023 Feb 27;61(2):159–73. doi: 10.1007/s12275-023-00015-x (PMC9998320; doi:10.1007/s12275-023-00015-x)
Supplement: Supplementary file 1 — Supplementary file1 (PDF 312 kb) [file 12275_2023_15_MOESM1_ESM.pdf]

Fig. S1. Molecular phylogeny of the lichen species in Maxwell Bay region. The trees were obtained by Maximum Likelihood method based on ITS rDNA. Additional trees were generated by Maximum Parsimony and Neighbor joining methods, the respective support values (ML/NJ/MP) are noted. Branches supported with bootstrap values > 70 and maintained by every method are indicated in thick. The specimens identified using molecular data in this paper are bold letters.

(A) Baeomycetales, Pertusariales & Acarosporales

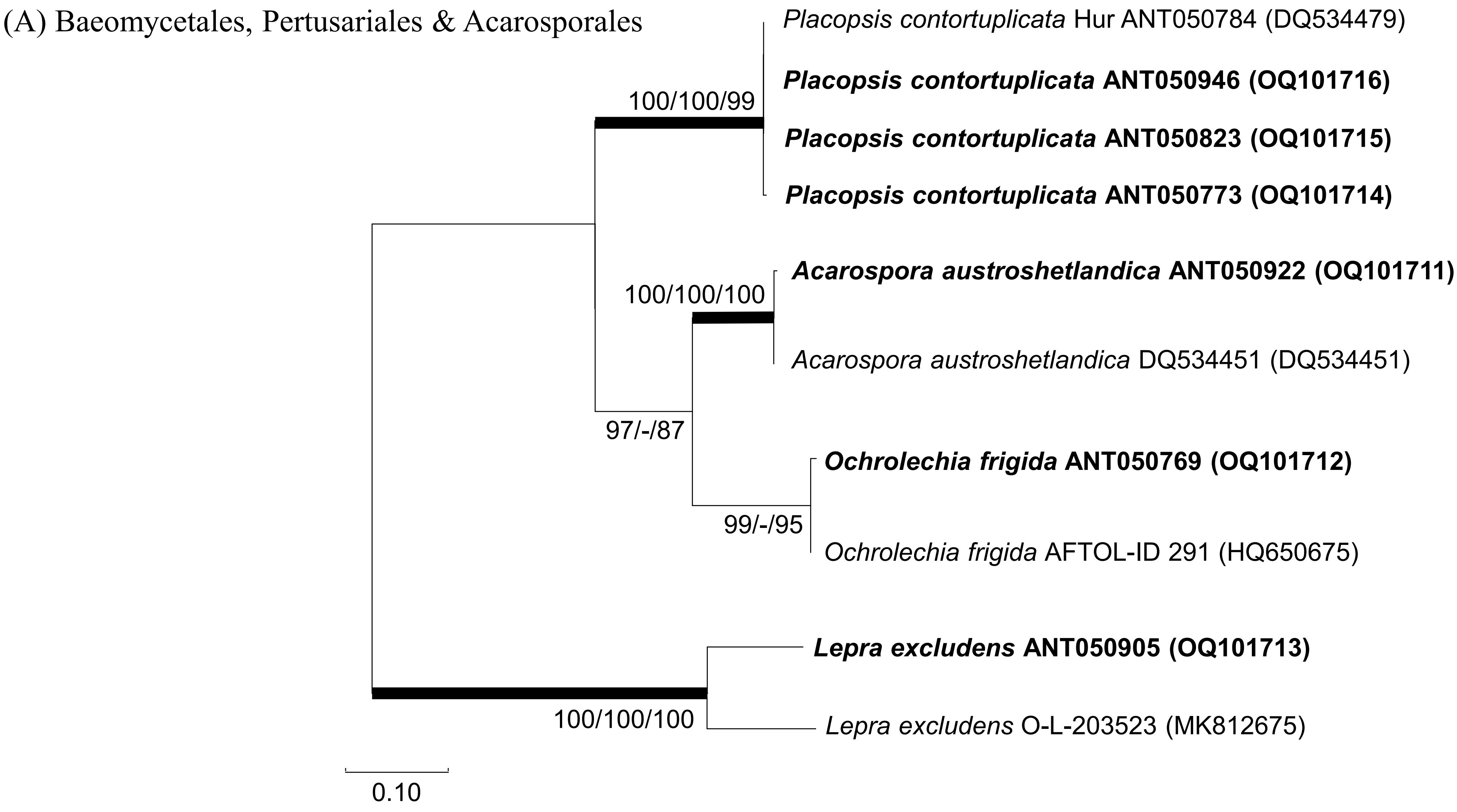

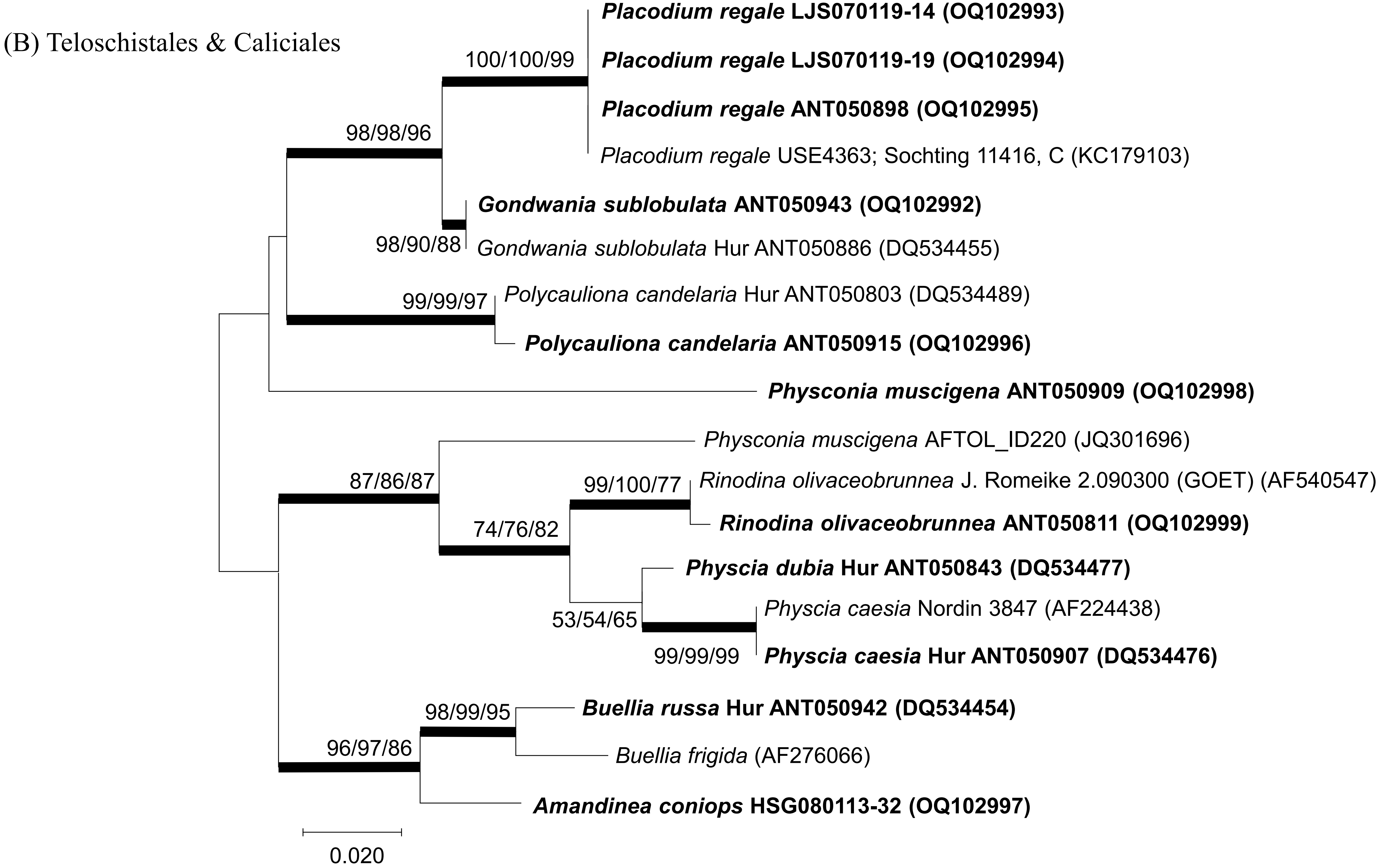
(B) Teloschistales & Caliciales
Placodium regale LJS070119-14 (OQ102993)
Placodium regale LJS070119-19 (OQ102994)
Placodium regale ANT050898 (OQ102995)
Placodium regale USE4363; Sochting 11416, C (KC179103)
100/100/99
98/98/96
Gondwania sublobulata ANT050943 (OQ102992)
Gondwania sublobulata Hur ANT050886 (DQ534455)
98/90/88
Polycauliona candelaria Hur ANT050803 (DQ534489)
Polycauliona candelaria ANT050915 (OQ102996)
99/99/97
Physconia muscigena ANT050909 (OQ102998)
Physconia muscigena AFTOL_ID220 (JQ301696)
87/86/87
Rinodina olivaceobrunnea J. Romeike 2.090300 (GOET) (AF540547)
Rinodina olivaceobrunnea ANT050811 (OQ102999)
99/100/77
74/76/82
Physcia dubia Hur ANT050843 (DQ534477)
Physcia caesia Nordin 3847 (AF224438)
Physcia caesia Hur ANT050907 (DQ534476)
53/54/65
99/99/99
Buellia russa Hur ANT050942 (DQ534454)
Buellia frigida (AF276066)
98/99/95
Amandinea coniops HSG080113-32 (OQ102997)
96/97/86
0.020

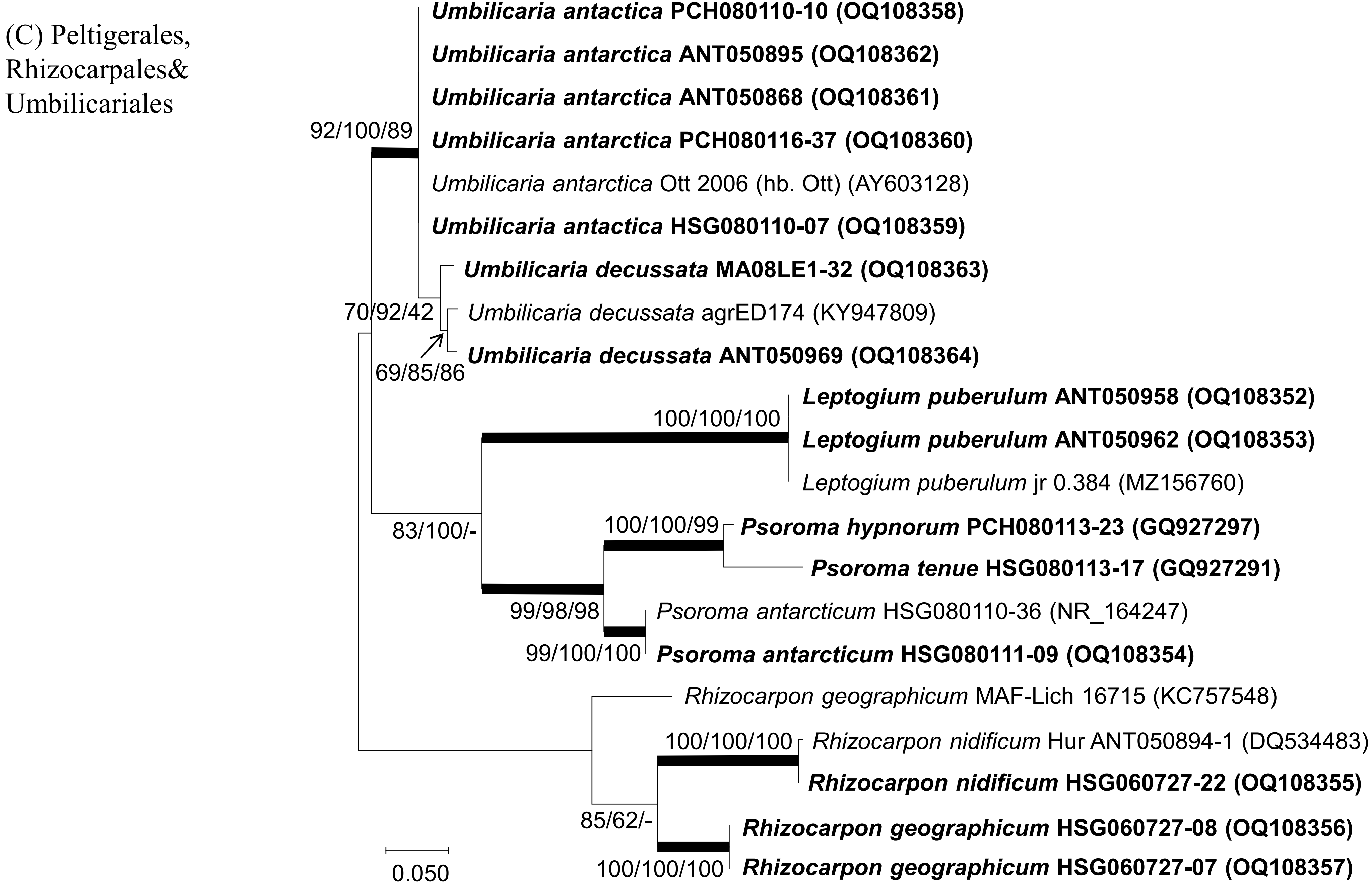

(C) Peltigerales, Rhizocarpales& Umbilicariales
Umbilicaria antactica PCH080110-10 (OQ108358)
Umbilicaria antarctica ANT050895 (OQ108362)
Umbilicaria antarctica ANT050868 (OQ108361)
Umbilicaria antarctica PCH080116-37 (OQ108360)
Umbilicaria antarctica Ott 2006 (hb. Ott) (AY603128)
Umbilicaria antactica HSG080110-07 (OQ108359)
Umbilicaria decussata MA08LE1-32 (OQ108363)
Umbilicaria decussata agrED174 (KY947809)
Umbilicaria decussata ANT050969 (OQ108364)
Leptogium puberulum ANT050958 (OQ108352)
Leptogium puberulum ANT050962 (OQ108353)
Leptogium puberulum jr 0.384 (MZ156760)
Psoroma hypnorum PCH080113-23 (GQ927297)
Psoroma tenue HSG080113-17 (GQ927291)
Psoroma antarcticum HSG080110-36 (NR_164247)
Psoroma antarcticum HSG080111-09 (OQ108354)
Rhizocarpon geographicum MAF-Lich 16715 (KC757548)
Rhizocarpon nidificum Hur ANT050894-1 (DQ534483)
Rhizocarpon nidificum HSG060727-22 (OQ108355)
Rhizocarpon geographicum HSG060727-08 (OQ108356)
Rhizocarpon geographicum HSG060727-07 (OQ108357)
92/100/89
70/92/42
69/85/86
100/100/100
83/100/-
100/100/99
99/98/98
99/100/100
100/100/100
85/62/-
100/100/100
0.050

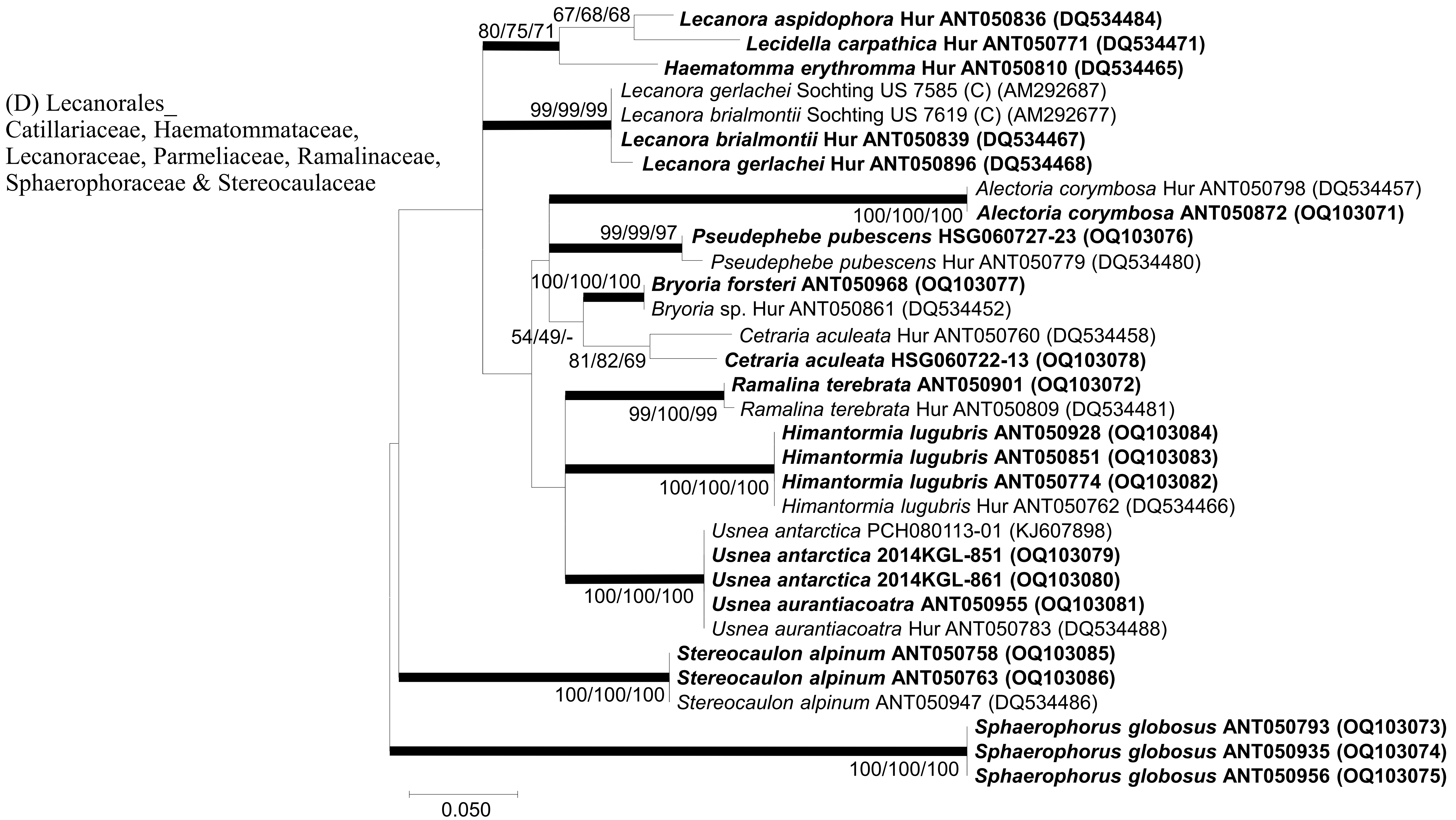
(D) Lecanorales_
Catillariaceae, Haematommataceae,
Lecanoraceae, Parmeliaceae, Ramalinaceae,
Sphaerophoraceae & Stereocaulaceae
67/68/68
80/75/71
Lecanora aspidophora Hur ANT050836 (DQ534484)
Lecidella carpathica Hur ANT050771 (DQ534471)
Haematomma erythromma Hur ANT050810 (DQ534465)
99/99/99
Lecanora gerlachei Sochting US 7585 (C) (AM292687)
Lecanora brialmontii Sochting US 7619 (C) (AM292677)
Lecanora brialmontii Hur ANT050839 (DQ534467)
Lecanora gerlachei Hur ANT050896 (DQ534468)
Alectoria corymbosa Hur ANT050798 (DQ534457)
100/100/100
Alectoria corymbosa ANT050872 (OQ103071)
99/99/97
Pseudephebe pubescens HSG060727-23 (OQ103076)
Pseudephebe pubescens Hur ANT050779 (DQ534480)
100/100/100
Bryoria forsteri ANT050968 (OQ103077)
Bryoria sp. Hur ANT050861 (DQ534452)
54/49/-
Cetraria aculeata Hur ANT050760 (DQ534458)
81/82/69
Cetraria aculeata HSG060722-13 (OQ103078)
Ramalina terebrata ANT050901 (OQ103072)
99/100/99
Ramalina terebrata Hur ANT050809 (DQ534481)
Himantormia lugubris ANT050928 (OQ103084)
Himantormia lugubris ANT050851 (OQ103083)
100/100/100
Himantormia lugubris ANT050774 (OQ103082)
Himantormia lugubris Hur ANT050762 (DQ534466)
Usnea antarctica PCH080113-01 (KJ607898)
Usnea antarctica 2014KGL-851 (OQ103079)
Usnea antarctica 2014KGL-861 (OQ103080)
100/100/100
Usnea aurantiacoatra ANT050955 (OQ103081)
Usnea aurantiacoatra Hur ANT050783 (DQ534488)
Stereocaulon alpinum ANT050758 (OQ103085)
Stereocaulon alpinum ANT050763 (OQ103086)
100/100/100
Stereocaulon alpinum ANT050947 (DQ534486)
Sphaerophorus globosus ANT050793 (OQ103073)
Sphaerophorus globosus ANT050935 (OQ103074)
100/100/100
Sphaerophorus globosus ANT050956 (OQ103075)
0.050

Table S1. List of taxa, collection source, and genes used in phylogenetic analyses

The specimens identified using molecular data and newly obtained sequences are indicated by bold letters.

No.	Taxon name	Source	Voucher specimen	GenBank accession no.
1	*Acarospora austroshetlandica*	Antarctica	ANT050835	DQ534451
2		Antarctica	**ANT050922**	**OQ101711**
3	*Alectoria corymbosa*	Antarctica	**ANT050872**	**OQ103071**
4		Antarctica	Hur ANT050798	DQ534457
5	*Amandinea coniops*	Antarctica	**HSG080113-32**	**OQ102997**
6	*Bryoria forsteri*	Antarctica	**ANT050968**	**OQ103077**
7	*Bryoria* sp.	Antarctica	Hur ANT050861	DQ534452
8	*Buellia frigida*	Antarctica	Unrecorded	AF276066
9	*B. russa*	Antarctica	**Hur ANT050942**	DQ534454
10	*Cetraria aculeata*	Antarctica	**HSG060722-13**	**OQ103078**
11		Antarctica	Hur ANT050760	DQ534458
12	*Cladonia carneola*	Antarctica	**PCH080116-34**	**OQ103109**
13		Antarctica	**PCH080116-36**	**OQ103108**
14		Finland	Stenroos 5583 (TUR)	AF454452
15	*C. chlorophaea*	Antarctica	**LYM100207-33**	**OQ103101**
16		Antarctica	**PCH080111-18**	**OQ103100**
17	*C. cornuta*	Antarctica	**2016KGIE-045**	**OQ103094**
18		Chile	Feuerer 60175a (TUR)	AF455196
19	*C. fimbriata*	Antarctica	**HSG080113-11**	**OQ103096**
20		Antarctica	**HSG080113-22**	**OQ103095**
21		USA	USA, 1997 Stenroos 5198 (TUR)	AF455220
22		Thailand	SP279 (RAMK)	EU113287
23	*C. galindezii*	Antarctica	HL090809-15B	OQ103102

No.	Taxon name	Source	Voucher specimen	GenBank accession no.
24	*C. gracilis*	Antarctica	**2016KGIE-006**	**OQ103093**
25		Antarctica	Hur ANT050816	DQ534462
26	*C. gracilis* (*C. furcata* in Kim *et al.* 2006)	Antarctica	**ANT050856**	EF489934
27		Antarctica	**ANT050857**	EF489935
28	*C. novochlorophaea*	Antarctica	**HSG080111-10**	**OQ103098**
29		Antarctica	**PCH110131-10**	**OQ103097**
30		Antarctica	**PCH110131-14**	**OQ103099**
31	*C. pleurota*	Antarctica	**ANT050858**	**OQ103106**
32		Antarctica	**PCH080124-16**	**OQ103107**
33	*C. pyxidata*	Antarctica	**Hur ANT050825**	DQ534463
34	*C. squamosa*	Sweden	1997 Stenroos 5120 (TUR)	AF457886
35		Antarctica	**2016KGIE-039**	**OQ103110**
36		Antarctica	**HSG091112-23**	**OQ103111**
37	*C. subulata*	Antarctica	**PCH080110-37**	**OQ103103**
38		Antarctica	**PCH110127-13**	**OQ103104**
39		Finland	Stenroos 5106 (TUR)	AF455180
40	*C. weymouthii*	New Caledonia	1998 Dennetiere 10 (H)	AF453689
41	*Cladonia* cf. *weymouthii*	Antarctica	**PCH110124-05**	**OQ103105**
42	*Gondwania sublobulata*	Antarctica	**ANT050943**	**OQ102992**
43		Antarctica	Hur ANT050886	DQ534455
44	*Haematomma erythromma*	Antarctica	**Hur ANT050810**	DQ534465
45	*Himantormia lugubris*	Antarctica	**ANT050774**	**OQ103082**
46		Antarctica	**ANT050851**	**OQ103083**
47		Antarctica	**ANT050928**	**OQ103084**
48		Antarctica	Hur ANT050762	DQ534466
49	*Lecanora aspidophora*	Antarctica	**Hur ANT050836**	DQ534484
50	*L. brialmontii*	Antarctica	**Hur ANT050839**	DQ534467

No.	Taxon name	Source	Voucher specimen	GenBank accession no.
51		Unrecorded	Sochting US 7619 (C)	AM292677
52	*L. gerlachei*	Antarctica	Hur ANT050896	DQ534468
53	*L. gerlachei*	Unrecorded	Sochting US 7585 (C)	AM292687
54	*Lecidella carpathica*	Antarctica	**Hur ANT050771**	DQ534471
55	*Lepra excludens*	Antarctica	**ANT050905**	**OQ101713**
56		Norway	O-L-203523	MK812675
57	*Leptogium puberulum*	Antarctica	**ANT050958**	**OQ108352**
58		Antarctica	**ANT050962**	**OQ108353**
59		Unrecorded	jr 0.384	MZ156760
60	*Ochrolechia frigida*	Unrecorded	AFTOL-ID 291	HQ650675
61		Antarctica	**ANT050769**	**OQ101712**
62	*Physcia caesia*	Antarctica	**Hur ANT050907**	DQ534476
63		Unrecorded	Nordin 3847	AF224438
64	*P. dubia*	Antarctica	**Hur ANT050843**	DQ534477
65	*Physconia muscigena*	Canada	AFTOL_ID220	JQ301696
66		Antarctica	**ANT050909**	**OQ102998**
67	*Placodium regale*	Antarctica	**ANT050898**	**OQ102995**
68		Antarctica	**LJS070119-14**	**OQ102993**
69		Antarctica	**LJS070119-19**	**OQ102994**
70		Antarctica	Sochting 11416	KC179103
71	*Placopsis contortuplicata*	Antarctica	**ANT050773**	**OQ101714**
72		Antarctica	**ANT050823**	**OQ101715**
73		Antarctica	**ANT050946**	**OQ101716**
74		Antarctica	Hur ANT050784	DQ534479
75	*Polycauliona candelaria*	Antarctica	Hur ANT050803	DQ534489
76		Antarctica	**ANT050915**	**OQ102996**
77	*Pseudephebe pubescens*	Antarctica	ANT050779	DQ534480

No.	Taxon name	Source	Voucher specimen	GenBank accession no.
78		Antarctica	**HSG060727-23**	**OQ103076**
79	*Psoroma antarcticum*	Norway	HSG080110-36	NR_164247
80	*Psoroma antarcticum*	Antarctica	**HSG080111-09**	**OQ108354**
81	*P. hypnorum*	Antarctica	**PCH080113-23**	GQ927297
82	*P. tenue*	Antarctica	**HSG080113-17**	GQ927291
83	*Ramalina terebrata*	Antarctica	**ANT050901**	**OQ103072**
84		Antarctica	Hur ANT050809	DQ534481
85	*Rhizocarpon geographicum*	Antarctica	**HSG060727-07**	**OQ108357**
86		Antarctica	**HSG060727-08**	**OQ108356**
87		Unrecorded	MAF-Lich 16715	KC757548
88	*R. nidificum*	Antarctica	**HSG060727-22**	**OQ108355**
89		Antarctica	Hur ANT050894-1	DQ534483
90	*Rinodina olivaceobrunnea*	Antarctica	**ANT050811**	**OQ102999**
91		Antarctica	J. Romeike 2.090300 (GOET)	AF540547
92	*Sphaerophorus globosus*	Antarctica	**ANT050793**	**OQ103073**
93		Antarctica	**ANT050935**	**OQ103074**
94		Antarctica	**ANT050956**	**OQ103075**
95	*Stereocaulon alpinum*	Antarctica	**ANT050758**	**OQ103085**
96		Antarctica	**ANT050763**	**OQ103086**
97		Antarctica	ANT050947	DQ534486
98	*Umbilicaria antarctica*	Antarctica	**ANT050868**	**OQ108361**
99		Antarctica	**ANT050895**	**OQ108362**
100		Antarctica	**HSG080110-07**	**OQ108359**
101		Antarctica	Ott 2006 (hb. Ott)	AY603128
102		Antarctica	**PCH080110-10**	**OQ108358**
103		Antarctica	**PCH080116-37**	**OQ108360**
104	*U. decussata*	Unrecorded	agrED174	KY947809

No.	Taxon name	Source	Voucher specimen	GenBank accession no.
105		Antarctica	**ANT050969**	**OQ108364**
106		Antarctica	**MA08LE1-32**	**OQ108363**
107	*Usnea antarctica*	Antarctica	**2014KGL-851**	**OQ103079**
108		Antarctica	**2014KGL-861**	**OQ103080**
109		Antarctica	PCH080113-01	KJ607898
110	*U. aurantiaco-atra*	Antarctica	**ANT050955**	**OQ103081**
111		Antarctica	Hur ANT050783	DQ534488
